# Urinary insulin signaling pathway related proteins may serve as potential biomarkers for monitoring diabetes mellitus without hypertension and hyperlipidemia

**DOI:** 10.1097/MD.0000000000032862

**Published:** 2023-02-03

**Authors:** Man Zhao, Qian Meng, Man Zhang

**Affiliations:** a Clinical Laboratory Medicine, Beijing Shijitan Hospital, Capital Medical University, Beijing, China; b Beijing Key Laboratory of Urinary Cellular Molecular Diagnostics, Beijing, China; c Clinical Laboratory Medicine, Peking University Ninth School of Clinical Medicine, Beijing, China.

**Keywords:** diabetes, insulin signaling pathway, markers, proteomics, urine

## Abstract

The insulin signaling pathway plays an important role in the development of diabetes mellitus. The expression of insulin signaling pathway related proteins in the urine of diabetic patients has not been reported. The aim of this study was to analyze and verify the expression of insulin signaling pathway related proteins in the urine of diabetic patients without hypertension and hyperlipidemia, and to explore their clinical application value. Based on data-independent acquisition proteomics technology and bioinformatics, the urinary protein expression profile of diabetic patients without hypertension and hyperlipidemia was established. Western blot and enzyme-linked immunoassay were performed to verify the expression of insulin signaling pathway related proteins in the urine of diabetic patients. Sixteen proteins related to the insulin signaling pathway were screened in urine, and 7 of them were differentially expressed in the urine of diabetic patients without hypertension and hyperlipidemia. Further quantitative analysis showed that the downregulation of protein kinase CAMP-dependent type II regulatory subunit α, growth factor receptor bound protein 2, and guanine nucleotide-binding protein G(s) in the urine of diabetic patients without hyperlipidemia and hypertension was consistent with the preliminary screening results. In this exploratory study, we detected the expression of insulin signaling pathway related proteins in the urine of diabetic patients without hypertension and hyperlipidemia. protein kinase CAMP-dependent type II regulatory subunit α, growth factor receptor bound protein 2, and guanine nucleotide-binding protein G(s) in the urine of diabetic patients were downregulated, which was associated with diabetes. They may be promising noninvasive biomarkers for monitoring diabetes.

## 1. Introduction

Diabetes mellitus (DM) is a disorder of carbohydrate, protein and fat metabolism brought about by absolute or relative insufficiency of insulin secretion and impaired insulin utilization, characterized mainly by hyperglycemia. At present, diabetes is mainly diagnosed by detecting the glucose level in the blood, combined with patients’ clinical symptoms such as polydipsia, polyuria, and weight loss.^[1,2]^ Repeated venipuncture during monitoring can be painful and inconvenient for diabetic patients. Therefore, convenient, rapid and noninvasive biomarkers are of great importance for the early diagnosis of diabetes.

Urine is a body fluid that can be collected continuously and non-invasively. Urine is waste that is processed by the kidneys to filter and to some extent reflects the state of the blood and the body as a whole.^[3,4]^ The rapid development of proteomics technology has made urine proteomics an important research direction for various diseases.^[5,6]^

Insulin promotes glucose uptake and utilization and inhibits hepatic gluconeogenesis. It is scientifically researched to regulate glucose and lipid metabolism, lowering blood glucose to keep it at normal levels. The biological effects of insulin are achieved through a series of protein kinases and phosphatases.^[7–10]^ Any abnormality in the insulin signaling pathway may disrupt glucose metabolism, which in turn lead to the development of diabetes.^[11–13]^ Therefore, the study on the insulin signaling pathway related proteins in the urine of diabetic patients has attracted our attention.

In order to achieve early detection of diabetes, we hope to screen urine biomarkers that are convenient, rapid, and noninvasive. The objective of this study was to compare the differential expression of insulin signaling pathway related proteins in urine between healthy controls and diabetic patients without hypertension and hyperlipidemia. Their potential value as noninvasive biomarkers for diabetes monitoring was also explored.

## 2. Materials and methods

### 2.1. Patient and sample collection

The study was approved by the Ethics Committee of Beijing Shijitan Hospital, Capital Medical University and complied with the principles of the Declaration of Helsinki. The ethical approval number is SJTKY11-1X-2021 (115). All subjects agreed to the study content and signed an informed consent form. From December 2021 to May 2022, a total of 72 patients with DM were selected from Beijing Shijitan Hospital, Capital Medical University. A total of 73 age and sex matched healthy individuals were selected as normal controls (NC). According to World Health Organization diagnostic criteria, patients with fasting plasma glucose ≥ 7.0 mmol/L, glycosylated hemoglobin type A1c ≥ 6.5%, or Oral Glucose Tolerance Test 2-hour glucose ≥ 11.1 mmol/L were diagnosed as diabetic patients. Blood pressure and lipid levels of participants were within the normal reference range. Urinary diseases, tumors and autoimmune diseases were excluded from all subjects.

First clean mid-morning urine samples were collected from subjects who met the inclusion criteria. Submitted urine was collected and poured into tubes and labeled as requiring. Urine was centrifuged to remove sediment and stored in a −80°C refrigerator for use.

### 2.2. Mass spectrometry

Urine was analyzed by data-independent acquisition proteomics methods. Urine specimens were centrifuged at 2000 g for 10 minutes. The supernatant was removed and concentrated by adding urea pellets to fully dissolve and ultrafiltration in an ultrafiltration tube. The supernatant was sought and enzymatic desalting was performed on it. The isolated and purified protein extracts were digested enigmatically by multiplex digestion reaction into small molecule peptide fragments. The enzyme-digested supernatant was collected and placed in a previous centrifuge tube, vacuum lyophilized and stored at −20°C. After redissolving the lyophilized powder, the samples were centrifuged at 14,000 g for 20 minutes at 4°C. 1 μg of supernatant was put into the machine for testing. The mass spectrometer was a Q-EXactive HF-X (Thermo Fisher Scientific, MA). The ion source was a Nanospray Flex™ (NSI) (Thermo Fisher Scientific, MA) with an ion spray voltage of 2.4 kV. The scan resolution was m/z 350 to 1500. The maximum injection time was 45 ms. The peptide fragmentation collision energy was placed at 27% to generate raw data for mass spectrometric detection.

Bioinformatics methods were utilized to screen for differential proteins. Differential protein analysis was performed by Gene Ontology, Kyoto Encyclopedia of Genes and Genomes (KEGG) and clustering analysis. Fold changes > 1.5 and *P* < .05 were considered as significant differences. Protein interaction analysis was performed by STRING (University of Zurich in Switzerland, Bern, Switzerland).

### 2.3. Western blot

To further validate the expression of insulin signaling pathway related proteins, we selected 7 proteins (HSPA5, IGF2, protein kinase CAMP-dependent type II regulatory subunit α [PRKAR2A], COL1A1, growth factor receptor bound protein 2 [GRB2], MAPT, guanine nucleotide-binding protein G(s) [GNAS]) that were at the core of the network in the bioinformatics analysis for Western blot validation. Protein loading buffer was added proportionally and boiled at 100°C for 15 minutes. Urine protein samples were loaded onto an 8% sodium dodecyl sulfate-polyacrylamide gel and were electrotransferred to polyvinylidene fluoride (PVDF) membranes. PVDF membranes were sealed with 5% skim milk powder for 2 hours at room temperature. The membranes were incubated with the following primary antibodies: HSPA5 (1:1000; cat. no. ab21685; Abcam), IGF2 (1:1000; cat. no. ab262713; Abcam), PRKAR2A (1:500; cat. no. PA5-106470; Invitrogen), COL1A1 (1:2000; cat. no. PA5-86949; Invitrogen), GRB2 (1:3000; cat. no. ab247231; Abcam), MAPT (1:1000; cat. no. ab32057; Abcam) and GNAS (1:1000; cat. no. ab283266; Abcam) at 4˚C overnight. Subsequently, the membranes were incubated with the second antibody for 90 minutes at 37°C. PVDF membranes were washed with tris buffered saline + Tween buffer and detected by enhanced chemiluminescence. Integrated optical density value of each band was analyzed using Lane 1D gel analysis software (Beijing Saizhi Venture Technology Co., Ltd, Beijing, China).

### 2.4. Enzyme-linked immunoassay (ELISA)

The validation cohort consisted of 52 diabetic patients without hypertension and hyperlipidemia and 53 age-sex matched NC. The concentrations of insulin signaling pathway related proteins in urine were determined using ELISA kits from Wuhan Fine Biotechnology (Wuhan, China). An aliquot of 10 µg of the subject’s urine sample was added to the bottom of the plate wells after measuring the urine protein concentration. The HRP coupling reagent was added to each well and incubated at 37°C for 60 minutes. The well plates were washed with detergent and the chromogenic agent was added in the dark. The reaction was terminated by adding a termination solution to each well after 15 minutes. The standard curve was plotted so that the sample concentration could be calculated.

### 2.5. Statistical analysis

Depending on the distribution of the variables, the data were presented as mean ± standard deviation or median. Statistical analysis was performed using SPSS 21.0 (SPSS Software Development Co., Ltd, Chicago, IL) and GraphPad Prism 8.0 (GraphPad Software, San Diego, CA). Differences between groups were compared by independent samples *t* test. A receiver operating characteristic curve was plotted to evaluate its sensitivity and specificity. *P* < .05 was considered statistically significant (2-tailed).

## 3. Results

### 3.1. Study population characteristics

Table 1 showed the discovery cohort of the study consisting of urine samples from 20 diabetic patients (DM) and 20 NC. The validation cohort included urine samples from 52 DM and 53 NC. Body mass index, fasting plasma glucose and glycosylated hemoglobin type A1c were significantly higher in the DM group than in the NC group (*P* < .001).

**Table 1:** Demographic and clinical characteristics of diabetes mellitus and normal controls.

	Discovery cohort			Validation cohort		
	DM (n = 20) NC (n = 20)		*P* value	DM (n = 52) NC (n = 53)		*P* value
Age (yr)	52.41 ± 1.34	54.32 ± 1.07	.192	54.17 ± 1.52	52.88 ± 1.33	.237
BMI (kg/m^2^)	23.47 ± 1.22	20.16 ± 2.56	<.001	24.05 ± 1.76	21.22 ± 2.07	<.001
FPG (mmol/L)	8.09 ± 0.31	5.21 ± 0.45	<.001	9.88 ± 0.64	5.14 ± 0.89	<.001
HbAlc (%)	7.11 ± 1.02	5.24 ± 1.32	<.001	7.07 ± 0.98	5.56 ± 0.71	<.001
SBP (mm Hg)	102 ± 15	101 ± 11	.323	107 ± 11	103 ± 8	.412
DBP (mm Hg)	74 ± 11	76 ± 9	.117	72 ± 15	73 ± 12	.111
CHOL (mmol/L)	4.17 ± 0.34	4.32 ± 0.12	.158	4.22 ± 0.19	4.05 ± 0.19	.162
TG (mmol/L)	0.88 ± 0.12	0.79 ± 0.09	.363	0.82 ± 0.16	0.83 ± 0.13	.341
HDL (mmol/L)	1.14 ± 0.17	1.09 ± 0.35	.118	1.26 ± 0.32	1.32 ± 0.27	.102
LDL (mmol/L)	2.11 ± 0.45	2.09 ± 0.37	.221	2.14 ± 0.31	2.19 ± 0.11	.216

CHOL = cholesterol, DBP = diastolic blood pressure, DM = diabetes mellitus, FPG = fasting plasma glucose, HbAlc = glycosylated hemoglobin type A1c, HDL = high density lipoprotein, LDL = low density lipoprotein, NC = normal controls, SBP = systolic blood pressure, TG = triglyceride.

### 3.2. Profiling insulin signaling pathway related proteins in the urine of DM

**Figure 1.**
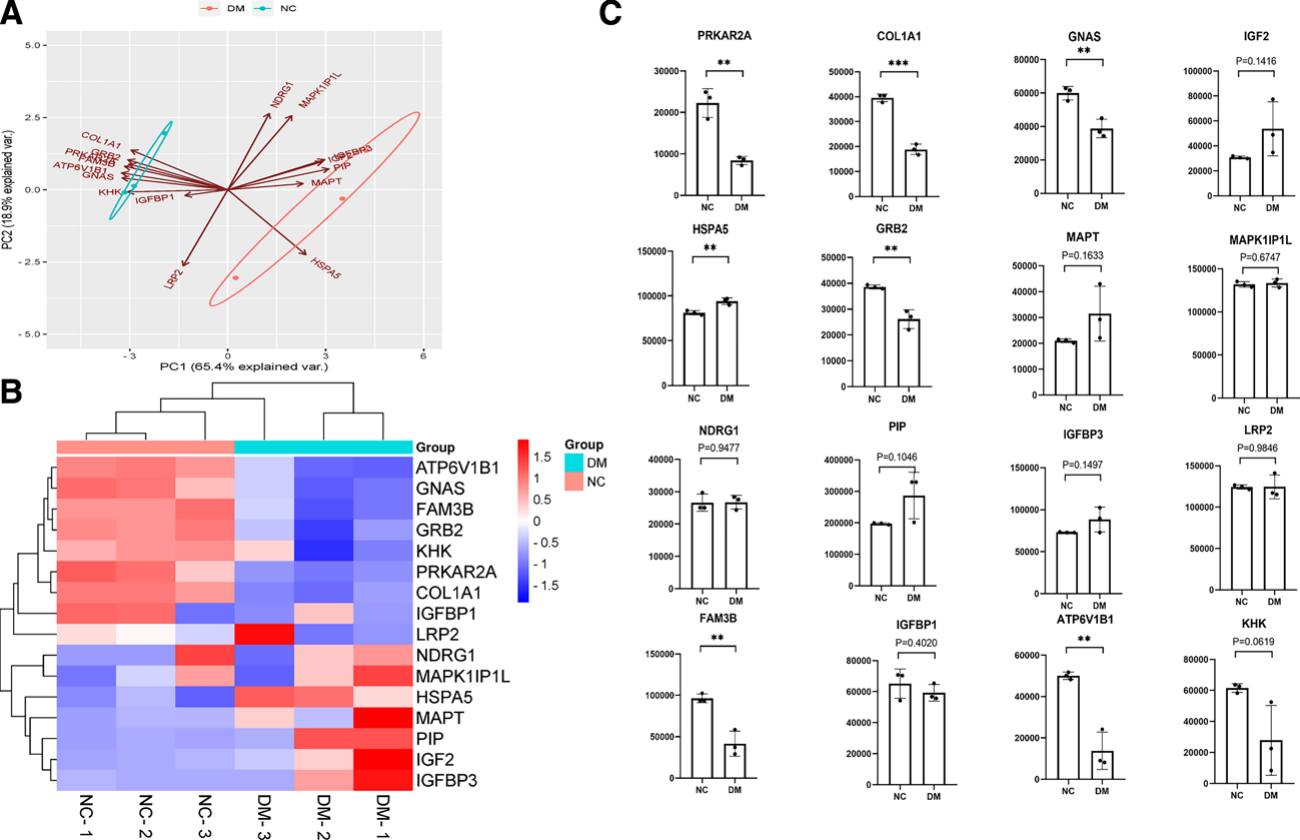
Proteomic analysis of insulin signaling pathway related proteins in urine. (A) PCA analysis of MS. (B) Cluster heat map analysis of insulin signaling pathway related proteins in DM and NC. (C) The abundance of insulin signaling pathway related proteins in the DM and NC. Data were mean ± SEM and significance was set at *P ≤ .05, **P ≤ .01, ***P ≤ .001. DM = diabetes mellitus, NC = normal controls, PCA = principal component analysis.

A comprehensive analysis of the urine proteomic profile was performed. Principal component analysis showed that the urine protein data were reliable and usable (Fig. 1A). The false discovery rate was .01 by searching the MaxQuant human database (Max Planck Institute, Munich, Germany) for insulin signaling pathway related proteins. The peptide sequence was obtained by theoretical enzyme digestion of all the protein sequences in database. Then the peptide sequence was theoretically splintered to form the spectrum. A total of 16 insulin signaling pathway related proteins were identified in the urine protein profile, as showed in the cluster heat map analysis (Fig. 1B). Fold changes > 1.5 and *P* < .05 were considered as significant differences. These proteins were differentially expressed in DM versus NC. In the DM group, PRKAR2A, COL1A1, GNAS, GRB2, FAM3B, and ATP6V1B1 were significantly downregulated and HSPA5 was upregulated. The relative abundance of these proteins in the DM and NC groups was presented in Figure 1C.

### 3.3. Bioinformatics analysis of potential urinary proteins

**Figure 2.**
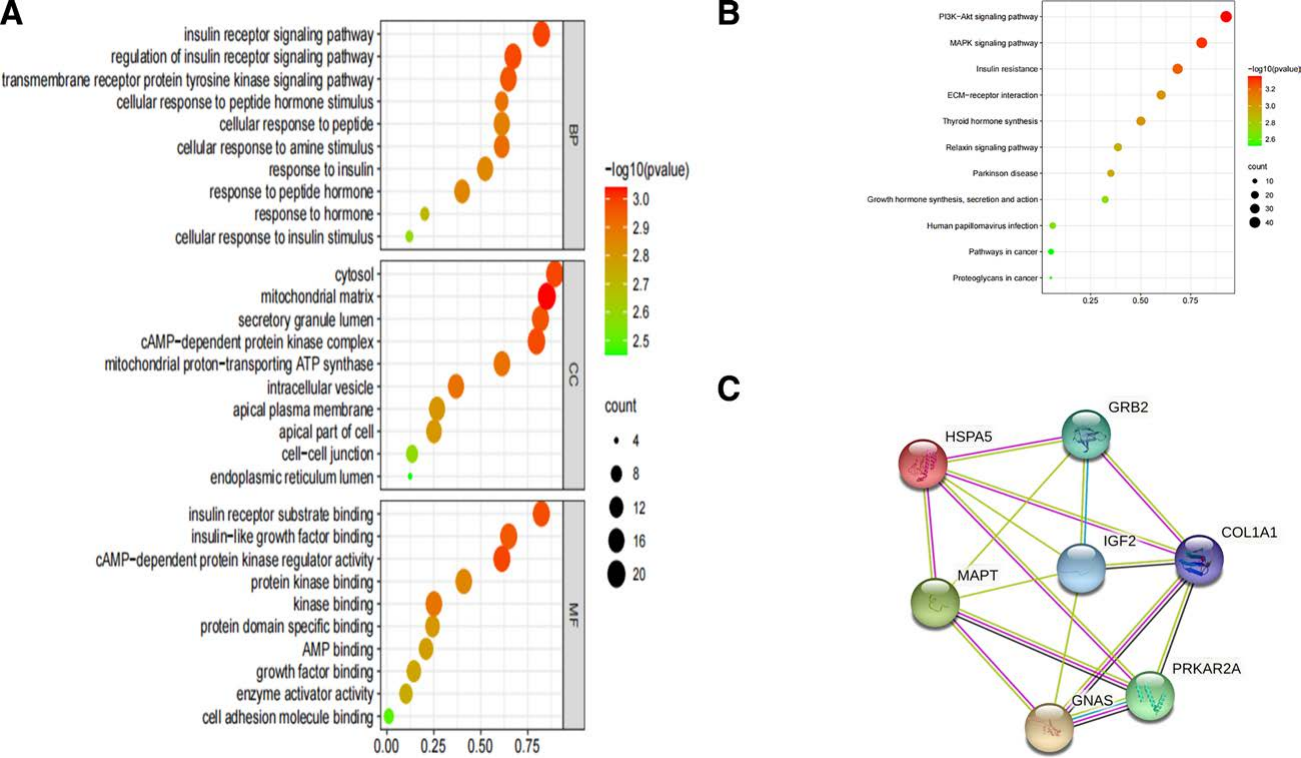
Functional analysis of insulin signaling pathway related proteins in urine. (A) GO enrichment analysis of 16 insulin signaling pathway related proteins. From top to bottom, biological process (BP), cellular component (CC), and molecular function (MF). (B) Sixteen insulin signaling pathway related proteins KEGG enrichment analysis. (C) Expression of insulin signaling pathway related protein by PPI network in DM and NC. DM = diabetes mellitus, GO = Gene Ontology, KEGG = Kyoto Encyclopedia of Genes and Genomes, PPI = protein-protein interaction, NC = normal controls.

To further understand the function of the 16 proteins, Gene Ontology and KEGG were performed by the Metascape platform (Metascape platform, WA). Sixteen proteins were entered into the Metascape database (http://metascape.org). The species was defined as Homo sapiens. Biological process analysis showed that these proteins were closely associated with the response to insulin, insulin receptor signaling pathway. Most of the cellular components were located in the endoplasmic reticulum lumen and their molecular functions were mainly insulin-like growth factor binding related, as shown in Figure 2A. The most abundant functions of PI3K-Akt signaling pathway, MAPK signaling pathway were analyzed by KEGG pathway, as shown in Figure 2B. In this study, we also performed interaction prediction analysis of 16 differential proteins by the STRING database. Proteins were input into the STRING data analysis platform (https://string-db.org) for network analysis of protein-protein interaction, with species set as “Homo sapiens.” HSPA5, IGF2, PRKAR2A, COL1A1, GRB2, MAPT and GNAS were found to be located on a core network with strong interactions between these proteins, as shown in Figure 2C.

### 3.4. Qualitative analysis of potential urinary proteins by Western blot

**Figure 3.**
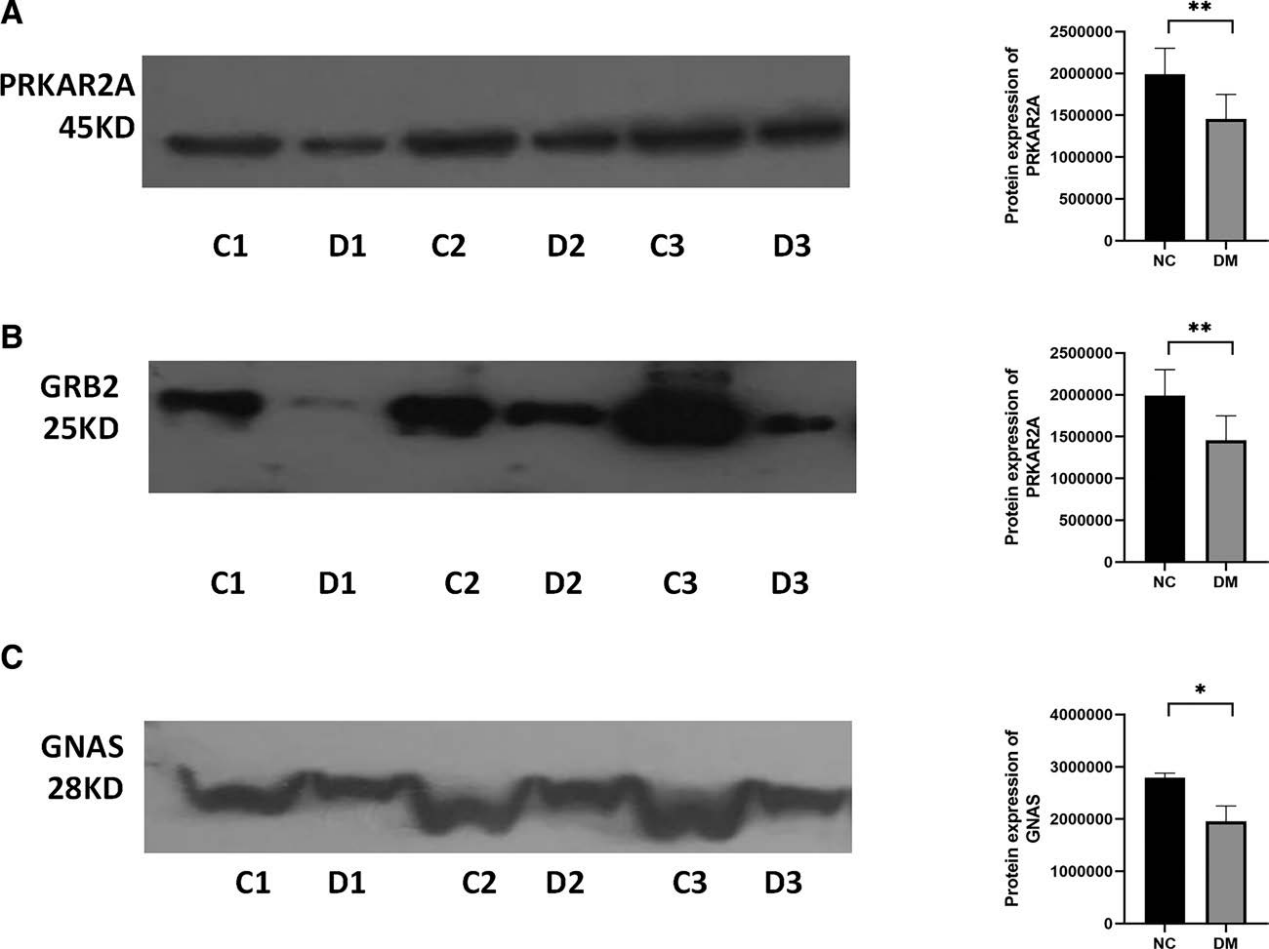
Analysis of Western blot in DM and NC. (A–C) The expression of PRKAR2A, GRB2, and GNAS by Western blot in different groups. All samples were independently analyzed 3 times in duplicate. (C1–C3) Western blot detection in urinary samples from NC. (D1–D3) Western blot detection in urinary samples from DM. DM = diabetes mellitus, GNAS = guanine nucleotide-binding protein G(s), GRB2 = growth factor receptor bound protein 2, NC = normal controls, PRKAR2A = protein kinase CAMP-dependent type II regulatory subunit α.

In order to verify the preliminary screening results of mass spectrometry, Western blot was used to verify the expression of insulin signaling pathway related proteins in the core network. PRKAR2A, GNAS and GRB2 were significantly downregulated in the DM compared to the NC (as shown in Fig. 3). The differences were statistically significant. Western Blot did not detect the expression of HSPA5, IGF2, MAPT and COL1A1. This may be related to the restriction of Western blot sensitivity.

### 3.5. Analysis of potential urinary proteins by ELISA

**Figure 4.**
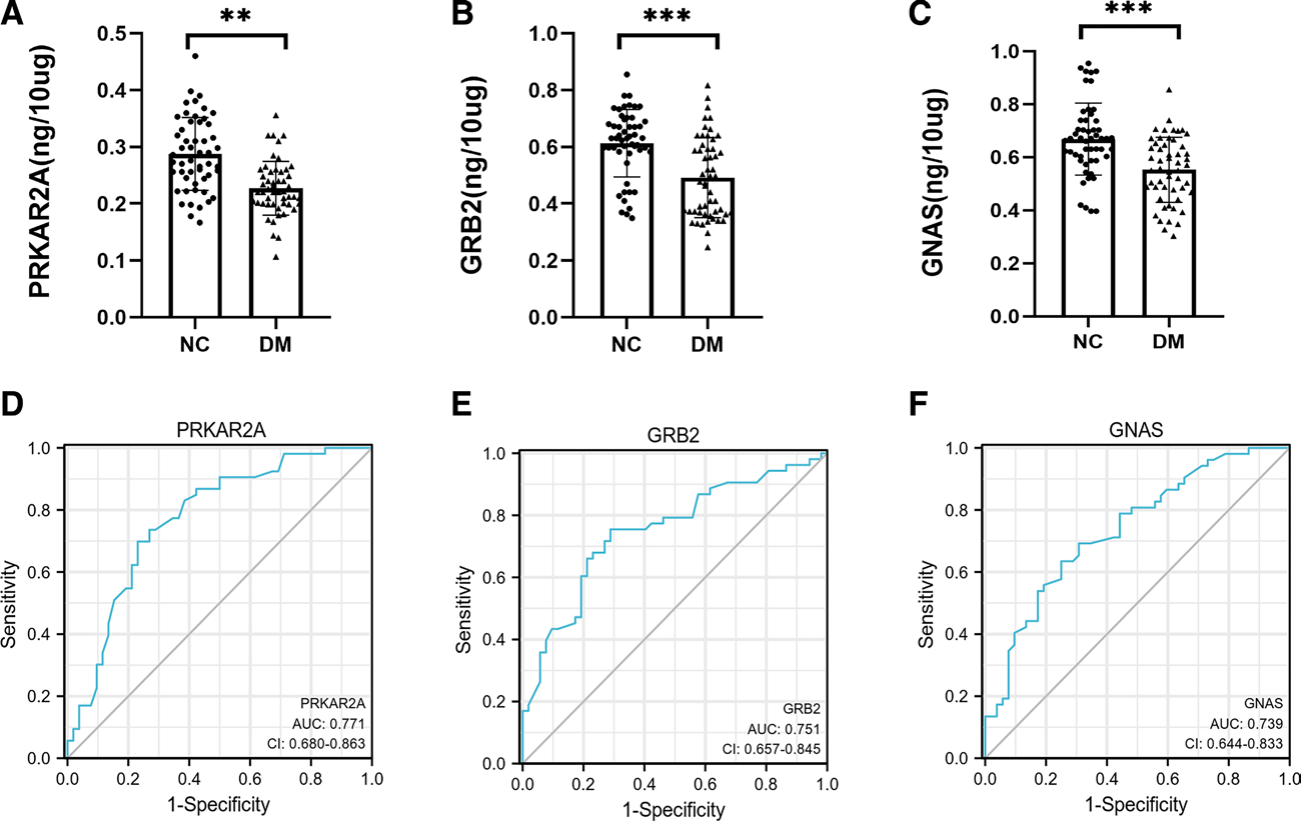
ELISA of diabetes mellitus (DM, n = 52) and normal control (NC, n = 53). (A–C) Expression of PRKAR2A, GRB2 and GNAS in NC and DM. The horizontal coordinate was the group and the vertical coordinate was the level strength of the protein. (D–F) ROC curve for monitoring urinary PRKAR2A, GRB2, and GNAS in diabetic patients. The unit was ng/10 mg, indicating the target protein content per 10 mg of total protein. ***, *P* < .001; **, *P* < .01. ELISA = enzyme-linked immunoassay, GNAS = guanine nucleotide-binding protein G(s), GRB2 = growth factor receptor bound protein 2, PRKAR2A = protein kinase CAMP-dependent type II regulatory subunit α, ROC = receiver operating characteristic.

Compared with the NC group, PRKAR2A, GRB2 and GNAS in the DM group were significantly downregulated (as shown in Fig. 4A–C). The difference was statistically significant. Based on the ELISA of 52 diabetic patients and 53 NC in the validation group, receiver operating characteristic curves were established (as shown in Fig. 4D–F). The areas under the curves for urinary PRKAR2A, GRB2, and GNAS were 0.771 (95% confidence interval [CI] = 0.680–0.863), 0.751 (95% CI = 0.657–0.845), and 0.739 (95% CI = 0.644–0.833), respectively. In conclusion, urinary insulin signaling pathway related proteins may be potential biomarkers for monitoring diabetes.

## 4. Discussion

The diagnosis of diabetes depends mainly on the glucose level in the blood and the clinical symptoms of the patient. The steps are tedious and complicated. Moreover, repeated blood collection has brought serious psychological pressure and economic burden to patients. To find a rapid, noninvasive and reliable biomarker, urine from diabetic patients without hypertension and hyperlipidemia was selected for proteomic study. The expression of insulin signaling pathway related proteins in the urine of diabetic patients has rarely been reported. In the present study we concentrated on the relationship between insulin signaling pathway related proteins and DM. Based on the bioinformatics analysis, we first screened the expression of insulin signaling pathway related proteins in the urine of diabetic patients without hypertension and hyperlipidemia, and then analyzed the function of these proteins. Then western blot and ELISA were utilized to verify their expression in urine. This helps to explore the influence of insulin signaling pathways on the pathogenesis of diabetes.

Insulin enters the cell membranes of target organs through the blood circulation and upregulates the expression of glucose transporter proteins. It increases the utilization of glucose by the organism, thus reducing glucose in the blood. Insulin mainly activates intracellular signaling pathways to monitor cell growth, metabolism and survival. Any abnormality in the insulin signaling pathway leads to a reduction in the biological effects of insulin and triggers disorders of glucolipid metabolism in the body. Previous studies related to the insulin signaling pathway have focused on tissues and other aspects.^[14–16]^ Chen Yanxing et al studied insulin signaling in brain tissues and concluded that insulin signaling pathway plays an important role in Alzheimer disease.^[17]^ Some researchers suggested that defective protein kinase A (PKA) activation in diabetic cardiomyocytes, partly mediated by lack of insulin signaling, lead to abnormal activation of primary metabolic regulators. It was believed that reduced insulin signaling would lead to the dysfunction of PKA response.^[18]^ However, the expression of insulin signaling pathway related proteins in the urine of diabetic patients has been less studied.

In this study, 16 proteins associated with the insulin signaling pathway in the urine of diabetic patients were screened by mass spectrometry. Further western blot and ELISA showed that urinary PRKAR2A, GRB2, and GNAS were downregulated in diabetic patients. This was consistent with the expression detected by mass spectrometry.

PRKAR2A is a major regulatory subunit of cyclic AMP dependent protein kinases that catalyzes downstream substrate protein phosphorylation by detecting cyclic AMP signaling.^[19–24]^ It is essential for β-cell differentiation and insulin secretion, and is involved in the regulation of lipid and glucose metabolism.^[25–27]^ Growth factor receptor binding protein 2 (GRB2) is a widely studied bridging protein involved in cell signaling. It is involved in the regulation of various downstream intracellular receptors by activating the Ras signaling pathway and forms a signaling cascade response upon activation. This ultimately leads to phosphorylation of the insulin receptor substrate serine, which ultimately leads to insulin resistance.^[28,29]^ GNAS is a key gene in the insulin secretion ability of β cells and an important signal transduction protein. It mainly acts on G protein-coupled receptor signaling pathway to activate adenylate cyclase, leading to increased cAMP and activation of PKA. It causes a cascade of amplified reactions, such as glycogen breakdown and elevated blood glucose level.^[30–32]^

In summary, differential expression of urinary insulin signaling pathway related proteins may be associated with diabetes. However, the mechanism of action needs to be further explored. The application of insulin signaling pathway related proteins in urine has the unique advantages of being noninvasive, rapid and easy. In this study, we analyzed the differential expression of insulin signaling pathway related proteins in urine between diabetic patients and NC without hypertension and hyperlipidemia. We detected significant downregulation of insulin signaling pathway related proteins PRKAR2A, GRB2, and GNAS in the urine of diabetic patients. It was helpful to explore the pathogenesis of diabetes from the perspective of insulin signaling pathway. They may be useful markers for diabetes monitoring and may serve as clinical targets for future diabetes treatment.

There are some limitations to this study. First, the samples in the current experiment were small, so more samples are planned for further evaluation and validation in later experiments. Second, due to the limitations of the mass spectrometry methodology, there may be some proteins that were not detected and missed. In addition, the specific mechanism of these insulin signaling pathway related proteins needs to be further studied. In subsequent experiments, we will increase the number of samples and use multiple experimental approaches to continue to focus on insulin signaling pathway related proteins in the urine of diabetic patients. Their potential value as biomarkers will be analyzed from multiple perspectives.

## Author contributions

**Funding acquisition:** Man Zhang.

Investigation: Qian Meng.

Writing – original draft: Man Zhao.

Writing – review & editing: Man Zhang.
